# JAK-STAT6 Pathway Inhibitors Block Eotaxin-3 Secretion by Epithelial Cells and Fibroblasts from Esophageal Eosinophilia Patients: Promising Agents to Improve Inflammation and Prevent Fibrosis in EoE

**DOI:** 10.1371/journal.pone.0157376

**Published:** 2016-06-16

**Authors:** Edaire Cheng, Xi Zhang, Kathleen S. Wilson, David H. Wang, Jason Y. Park, Xiaofang Huo, Chunhua Yu, Qiuyang Zhang, Stuart J. Spechler, Rhonda F. Souza

**Affiliations:** 1 Esophageal Diseases Center, Veterans Affairs North Texas Health Care System and University of Texas Southwestern Medical Center, Dallas, Texas, United States of America; 2 Department of Pediatrics, Children’s Health Children’s Medical Center, Dallas, Texas, United States of America; 3 Department of Pediatrics, University of Texas Southwestern Medical Center, Dallas, Texas, United States of America; 4 Medical Services, Veterans Affairs North Texas Health Care System, Dallas, Texas, United States of America; 5 Department of Internal Medicine, University of Texas Southwestern Medical Center, Dallas, Texas, United States of America; 6 Department of Pathology, University of Texas Southwestern Medical Center, Dallas, Texas, United States of America; 7 Eugene McDermott Center for Human Growth and Development, University of Texas Southwestern Medical Center, Dallas, Texas, United States of America; 8 Harold C. Simmons Comprehensive Cancer Center, University of Texas Southwestern Medical Center, Dallas, Texas, United States of America; 9 Department of Pathology, Children’s Health Children’s Medical Center, Dallas, Texas, United States of America; Baylor University Medical Center, UNITED STATES

## Abstract

**Background:**

Although most studies on treatments for eosinophilic esophagitis (EoE) have focused on effects in the epithelium, EoE is a transmural disease. Eosinophils that infiltrate the subepithelial layers of the esophagus lead to fibrosis and the serious complications of EoE, and current therapies have shown minimal effects on this fibrosis. We aimed to elucidate T helper (Th)2 cytokine effects on esophageal fibroblasts and to explore potential fibroblast-targeted therapies for EoE.

**Methods:**

We established telomerase-immortalized fibroblasts from human esophageal biopsies. We stimulated these esophageal fibroblasts with Th2 cytokines, and examined effects of omeprazole and inhibitors of the Janus kinase (JAK)—signal transducer and activator of transcription (STAT6) pathway (AS1517499, leflunomide, and ruxolitinib) on STAT6 phosphorylation, STAT6 nuclear translocation, and eotaxin-3 expression. We also measured the effects of these inhibitors in esophageal epithelial cells stimulated with Th2 cytokines.

**Results:**

As in esophageal epithelial cells, Th2 cytokines increased STAT6 phosphorylation, STAT6 nuclear translocation, eotaxin-3 transcription and protein secretion in esophageal fibroblasts. Unlike in epithelial cells, however, omeprazole did not inhibit cytokine-stimulated eotaxin-3 expression in fibroblasts. In contrast, JAK-STAT6 pathway inhibitors decreased cytokine-stimulated eotaxin-3 expression in both fibroblasts and epithelial cells.

**Conclusions:**

Omeprazole does not inhibit Th2 cytokine-stimulated eotaxin-3 expression by esophageal fibroblasts, suggesting that PPIs will have limited impact on subepithelial EoE processes such as fibrosis. JAK-STAT6 pathway inhibitors block Th2 cytokine-stimulated eotaxin-3 expression both in fibroblasts and in epithelial cells, suggesting a potential role for JAK-STAT inhibitors in treating both epithelial inflammation and subepithelial fibrosis in EoE.

## Introduction

Eosinophilic esophagitis (EoE) is an immunologic disorder with manifestations mediated by Th2 cytokines such as interleukin (IL)-4 and IL-13.[1] Eosinophils accumulate in the esophagus of EoE patients when Th2 cytokines stimulate signal transducer and activator of transcription (STAT)6 signaling in esophageal epithelial cells, causing them to produce eotaxin-3, a potent eosinophil chemoattractant that draws circulating eosinophils into the esophagus.[2, 3] Most reports involving the histology of EoE have focused on epithelial findings in esophageal pinch biopsy specimens. However, the few reports available on histologic findings in EoE esophagectomy specimens have described transmural involvement, with eosinophils infiltrating all layers of the esophagus from epithelium to adventitia.[4] Within the esophagus, this eosinophilic infiltration contributes to tissue remodeling with the development of fibrosis in the lamina propria (subepithelial fibrosis) and deeper layers that results in problematic mucosal fragility, rings and strictures characteristic of EoE.[5–9] A number of studies have shown that the esophageal epithelial eosinophilia of EoE responds to topical steroids and diet therapy, but only recently have a few studies explored the efficacy of these treatments for reversing esophageal fibrosis.[10–16] Furthermore, most studies focus on the effects of these treatments on epithelial cells and not on fibroblasts.[17, 18] Thus, studies exploring the effects of Th2 cytokines on fibroblasts may identify novel therapeutic targets at which to direct agents to treat patients with fibrosis in EoE.

Some 30% to 50% of patients who have esophageal eosinophilia and symptoms typical of EoE respond to treatment with proton pump inhibitors (PPIs).[19–22] This condition has been called PPI-responsive esophageal eosinophilia (PPI-REE).[1] Recent studies have shown that the clinical, endoscopic, histologic and esophageal gene expression features of PPI-REE and EoE are virtually identical, and multivariate analyses have not identified any feature that can distinguish PPI-REE from EoE.[21, 23, 24] Thus, available evidence suggests that PPI-REE and EoE are similar, if not identical disorders.[25] In earlier studies, we showed that the PPI omeprazole blocked STAT6 from binding to the eotaxin-3 promoter in esophageal epithelial cells, thereby preventing Th2 cytokines from stimulating eotaxin-3 expression.[2, 3] We found that this anti-inflammatory effect of omeprazole was entirely independent of its effects on gastric acid secretion, and we suggested that PPI inhibition of Th2 cytokine-stimulated esophageal secretion of eotaxin-3 might be the mechanism underlying PPI-REE. Those studies used esophageal epithelial cells from EoE patients, and a number of studies by other investigators have documented that epithelial eosinophilia in the esophagus can respond to PPI therapy. However, we are aware of only one clinical study that explored the effect of PPIs on esophageal fibrosis in patients with esophageal eosinophilia, and that study identified no effect of these agents on subepithelial fibrosis.[26]

In our present study, we have developed and characterized human, telomerase-immortalized, esophageal fibroblast cell lines in order to elucidate Th2 cytokine effects on esophageal fibroblasts and to explore potential fibroblast-targeted therapies for fibrosis associated with esophageal eosinophilia. Unlike in esophageal epithelial cells, we show that omeprazole does not block Th2 cytokine-induced eotaxin-3 expression by these fibroblasts. In contrast, we found that inhibitors of Janus kinase (JAK)-STAT6 pathway (AS1517499, leflunomide, and ruxolitinib) can block Th2 cytokine-induced eotaxin-3 expression in esophageal fibroblasts as well as in esophageal epithelial cells. Our findings suggest a potential therapeutic role for JAK-STAT6 pathway inhibitors, which conceivably could treat both the epithelial inflammation and subepithelial fibrosis in patients with EoE.

## Materials and Methods

### Isolation and culture of primary human esophageal fibroblasts

From esophageal mucosal biopsy specimens taken during endoscopic examinations in two patients, we established two esophageal fibroblast cell lines with the subjects’ written informed consent. One line (FEE4-T) was from a man with EoE who had a history of dysphagia and food impactions, an endoscopy showing vertical furrows and multiple fine rings in the esophagus, and esophageal biopsy specimens showing ≥15 eosinophils per high power field with numerous eosinophilic microabscesses. The other fibroblast cell line (BEF-T) was from a man with long-segment Barrett’s esophagus. Establishment of the cell lines from our patients’ biopsy specimens was performed as previously described.[27–29] The establishment of esophageal fibroblast cell lines from human subjects was approved by the Dallas VA Medical Center Institutional Review Board.

To establish primary cultures, 12 biopsy samples of the distal esophageal mucosa were taken with jumbo biopsy forceps (Olympus FB-50K-1; Olympus, Tokyo, Japan). Biopsy samples were placed in cold Hank’s salt solution (Mediatech, Manassas, VA) containing 1% antibiotic-antimycotic (Invitrogen, Carlsbad, CA) and processed within 2 hours of collection. Six esophageal mucosal biopsy samples were placed into each of two conical tubes containing trypsin (Invitrogen) at 37°C for 30 minutes. Following trypsinization, biopsies were dispersed into a single-cell suspension in four parts Dulbecco’s modified Eagle’s medium to one part Medium 199 (Gibco, Carlsbad, CA) supplemented with 10% fortified bovine calf serum (Hyclone Laboratories, Logan, UT), and gentamicin (50 μg/mL). For subculturing, fibroblasts were trypsinized using 0.05% trypsin-EDTA (Gibco), resuspended in fresh medium, reseeded at a 1:8 split ratio, and grown at 37°C in 5% CO_2_ with fresh media changes three times weekly.

The number of population doublings per passage was calculated as log [(number of cells harvested / number of cells plated) / log2]. The population doubling time was calculated as time between passages / population doubling.

### Telomerase Infection and Activity

Cells at 30–50% confluence were infected twice with a retroviral vector containing human telomerase reverse transcriptase flanked by lox-P sites (hTERTloxP) in the presence of 4 μg/ml of Polybrene (Sigma-Aldrich, St. Louis, MO), at population doublings 10–16. The fibroblasts containing the hTERTloxp vector were selected by treatment with 500 ng/ml puromycin for 7–9 days. Telomerase activity was measured in cultured cells before and after the introduction of human telomerase reverse transcriptase with the TRAP-eze Telomerase Detection kit (Intergen, Burlington, MA) as previously described.[27] Telomerase-expressing cervical cancer-derived HeLa cells were used as positive controls. Lysis buffer (Cell Signaling Technology, Beverly, MA) was used as a negative control.

### Cell Lines and Culture

The human esophageal fibroblasts cell lines (FEE4-T and BEF-T) were cultured in Dulbecco’s modified Eagle’s medium and Medium 199 (4:1 ratio) supplemented with 10% fortified bovine calf serum and gentamicin (50 μg/mL). Human colon fibroblast CCD-18Co (ATCC CRL-1459) and human lung fibroblast MRC-5 (ATCC CCL-171) were cultured in Eagle's Minimum Essential Medium supplemented with 10% fetal bovine serum. For individual experiments, fibroblasts were equally seeded, maintained in full growth media, and kept in log phase growth until ready to use. We used two non-neoplastic, telomerase-immortalized, esophageal squamous cell lines (EoE1-T and EoE2-T) that were created by our laboratory using esophageal mucosal biopsy specimens from patients who had EoE, as previously described.[2] Cells were maintained in monolayer culture at 37°C in humidified air with 5% CO2 in growth medium co-cultured with a fibroblast feeder layer as previously described.[30] For individual experiments, epithelial cells were equally seeded into standard culture dish without fibroblast feeder cells and maintained in growth medium.

### Immunofluorescence Staining

Cells were seeded at a density of 2 × 10^5^ cells per well onto glass cover slips and cultured in 6-well plates. Cells were fixed in 4% paraformaldehyde for 15 minutes, permeabilized for 10 minutes, and incubated with primary antibodies against fibroblast surface protein (1:200 dilutions, Abcam, Cambridge, MA) or pan-cytokeratin (1:200 dilution, Sigma-Aldrich) for 1 hour at room temperature. After 3 washes in phosphate-buffered saline, cells were incubated with Alexa Fluor 488—conjugated donkey-anti-mouse IgG (1:500, Invitrogen, Carlsbad, CA) for 1 hour at room temperature, and then counterstained with 4’,6-diamidino-2-phenylindole (DAPI). Images were recorded with a fluorescence microscope (DM6000B, Leica Microsystem Ltd., Heerbrugg, Switzerland).

### Nuclear/Cytoplasmic Fractionation and Western Blotting

Whole cells were lysed in 1x cell lysis buffer (Cell Signaling Technology, Danvers, MA). Nuclear extracts were isolated after 20 minutes of IL-13 or IL-4 stimulation using the NE-PER Nuclear and Cytoplasmic Extraction kit (Thermo Fisher Scientific, Rockford, IL) per manufacturer’s instructions. Protein concentrations were determined using the BCA-200 Protein Assay kit (Pierce, Rockford, IL). After separation and transfer to nitrocellulose membranes, the membranes were incubated with primary antibodies to α smooth muscle actin (1:1,000 dilutions, Sigma-Aldrich), fibronectin (1:1,000 dilutions, Santa Cruz, Dallas, TX), vimentin (1:1,000 dilutions, Santa Cruz), p53 (1:1,000 dilutions, Calbiochem, San Diego, CA), phospho-STAT6 Tyr641 (1:1,000 dilutions, Cell Signaling Technology, Beverly, MA), total STAT6 (1:1,000 dilutions, Cell Signaling Technology), β-tubulin (1:2,000 dilutions, Sigma-Aldrich), β-actin (1:2,000 dilutions, Sigma-Aldrich), or TFIID (1:500 dilutions, Cell Signaling Technology). Horseradish peroxidase secondary antibodies were used, and chemiluminescence was determined using the ECL Western blotting Substrate or the Super Signal West Dura detection system (Pierce). The proteins of interest were measured and normalized to respective loading control protein by densitometry (ImageJ National Institutes of Health, Bethesda MD).

### Anchorage-dependent Cell Growth Assays

An *in vitro* marker for transformed cells is the ability to grow in soft agar. A 12-well soft agar plate was created as previously described.[30] Five thousand telomerase-immortalized fibroblasts were plated in triplicate; OE33 esophageal adenocarcinoma cells (Sigma-Aldrich) served as a positive control. Plates were examined daily for 3 weeks. Cells were imaged using a Bio-Rad Molecular Imager (Bio-Rad, Hercules, CA).

### UV-B Irradiation

Telomerase-immortalized fibroblast lines were cultured overnight in 100 mm plates. Cells were rinsed several times with 1X phosphate buffered saline and exposed to 200 J/m^2^ UV-B irradiation as previously described.[27] Protein was harvested at 24 and 48 hours after irradiation. Non-irradiated cells served as controls.

### Cytogenetic analysis

Cytogenetic analysis was performed on esophageal fibroblast cell lines. Dividing cells were harvested from cultures incubated without mitogen, and Trypsin G-banded using standard methods.[31] Briefly, metaphase cells were obtained by colcemid arrest followed by hypotonic treatment with pre-warmed 0.075M KCl. They were then fixed and washed in freshly made modified Carnoy's fixative (3:1 absolute methanol:glacial acetic acid), dropped onto pre-cleaned, wet microscope slides and air-dried. Cytogenetic abnormalities were classified according to the International System for Human Cytogenetic Nomenclature.

### 3D Organotypic Culture

Organotypic cultures were performed using the protocol described by Kalbis et al.[32] Briefly, acellular collagen matrices using bovine type I collagen (Advanced BioMatrix, Carlsbad CA) were created on the bottom of 24mm transwell inserts (Sigma-Aldrich). To prepare the cellular collagen matrices, esophageal fibroblasts were suspended in a collagen mixture at a density of 5 x 10^4^ cells/ml. The fibroblast-collagen matrices were cultured for 7 days in fibroblast growth media. On day 2, the fibroblast-collagen matrices were released from the sides of the well to allow for matrix contraction. On day 7, esophageal epithelial cells EoE2-T were seeded on the surface of the fibroblast-collagen matrices at a density of 5 x 10^5^ cells/well in epidermalization growth media as per protocol.[32] On day 11, the organotypic cultures were raised to the air-liquid interface. On day 14, the cultures were harvested and fixed for 1hr in 10% buffered formalin phosphate before being paraffin-embedded and sectioned for hematoxylin and eosin staining.

### Stimulation or Treatment of Fibroblasts and Epithelial Cells

Cells were stimulated with 0–100 ng/ml of IL-13 or IL-4 (R&D Systems, Minneapolis, MN) for up to 48 hours. For PPI studies, omeprazole (Sigma-Aldrich) was acid-activated in medium with pH 5.5 for 30 minutes. Cells were then pre-treated for 2 hours with omeprazole (50μM) in neutral medium with pH 7.4 prior to the addition of Th2 cytokines. The PPI remained in the media throughout the period of cytokine stimulation. For JAK-STAT6 inhibition studies, cells were pre-treated for 30 minutes with leflunomide (Sigma-Aldrich), 30 minutes with AS1517499 (Axon Medchem, Groningen, The Netherlands), or 2 hours with ruxolitinib (InvivoGen, San Diego, CA) prior to the addition of Th2 cytokines. The inhibitors remained in the media throughout the period of cytokine stimulation.

### Enzyme-linked Immunosorbent Assays (ELISA) for Eotaxin-3

We performed ELISA of conditioned media to assess the secretion of eotaxin-3 by fibroblasts. Conditioned media collected were centrifuged to remove cellular debris. Cytokine concentrations were determined using commercially available ELISA kits (R&D Systems) per manufacturer’s instructions. The absorbance of each well was read at 450 nm and 540 nm using a DTX 880 Multimode plate reader (Beckman Coulter, Brea, CA).

### Qualitative Polymerase Chain Reaction (PCR)

Total RNAs were isolated from cell lines using RNeasy Mini kit (Qiagen, Valencia, CA) per manufacturer’s instructions and quantitated by spectrophotometry. Reverse transcription was performed using QuantiTect Reverse Transcription kit (Qiagen) per manufacturer’s instructions. PCR was performed for eotaxin-3 mRNA. The primer sequences and PCR products sizes for qualitative analyses were as follows:

1. Eotaxin-3 forward 5’-AACTCCGAAACAATTGTACTCAGCTG-3’ and Eotaxin-3 reverse 5’-GTAACTCTGGGAGGAAACACCCTCTCC-3’, 151 bp;
2. GAPDH forward 5’ TCCCACCTTTCTCATCCAAG-3’ and GAPDH reverse 5’-GTCTGCAAAAGGAGTGAGGC-3, 194 bp.

PCR conditions consisted of 94°C for 5 minutes followed by 30 cycles at 94°C for 30 seconds, 55°C for 30 seconds, and 72°C for 30 seconds. After amplification, PCR products were electrophoresed on 2% agarose gels and stained with ethidium bromide. GAPDH transcripts served as internal controls. The transcripts of interest were measured and normalized to GAPDH by densitometry (NIH ImageJ).

### Chromatin Immunoprecipitation (ChIP) Assay

Cells were pre-treated for 24 hours with acid-activated omeprazole (50 μM) in medium with pH 7.4 prior to the addition of IL-4 (1 ng/ml). Cells were stimulated with IL-4 in the presence or absence of omeprazole, or control medium for 2 hours. ChIP assay was performed using Qiagen EpiTect ChIP OneDay Kit reagents as per protocol. In brief, cells were cross-linked with 1% formaldehyde. After quenching the reaction, the cells were harvested and lysed in lysis buffer with protease inhibitor cocktail. To shear the chromatin, lysates were sonicated to generate DNA fragments of 0.5–1 kilobase. The sheared chromatin was centrifuged, and each aliquot of ChIP ready chromatin (IP fraction) was pre-cleared with Protein A-agarose beads for 50 minutes. 1% of each IP fraction was set aside as the Input Fraction for later DNA isolation and purification. Each IP fraction (1 ml) was immunoprecipitated overnight at 4°C with 4 μg of polyclonal rabbit anti-human STAT6 (Santa Cruz, sc621). Rabbit IgG 2 μg (Cell SignalingTechnology, 2729S) was used as isotype control. For immunoprecipitation, each IP fraction was incubated with Protein A-agarose beads on a rotator at 4°C for 1 hour. The beads were washed 5 times with wash buffer. DNA was isolated using 100 μl of 10% Chelex 100 followed by boiling for 10 minutes and collecting and pooling supernatants. DNA was purified using DNA spin columns. For qualitative PCR, 5 μl of purified DNA template was used in a 25 μl reaction with the eotaxin-3 forward primer 5’-GTGCTGCTTCTGTTCCCAACCACA-3’ and the eotaxin-3 reverse primer 5’-ACTCCTGCCTGATCCCCTT-3’ spanning nucleotides -82 to +6 within the eotaxin-3 promoter to assess for STAT6 binding. PCR conditions consisted of 94°C for 5 minutes followed by 40 cycles at 94°C for 30 seconds, 60°C for 30 seconds, and 72°C for 30 seconds. After amplification, PCR products were electrophoresed on 2% agarose gels and stained with ethidium bromide. PCR products were measured and normalized to Input Fraction by densitometry (NIH ImageJ).

### Statistical Analyses

Data were collected from at least two independent experiments. Quantitative data were expressed as means ± standard error of the mean (SEM). Statistical analyses were performed using one-way analysis of variance followed by Bonferroni method to correct for multiple comparisons. Dunnett method was used when all comparisons were made against one condition (GraphPad Software, San Diego, CA). *P* values ≤ 0.05 were considered significant for all analyses.

## Results

### Establishment and Characterization of Telomerase-Immortalized Esophageal Fibroblast Cell Lines

**Fig 1:**
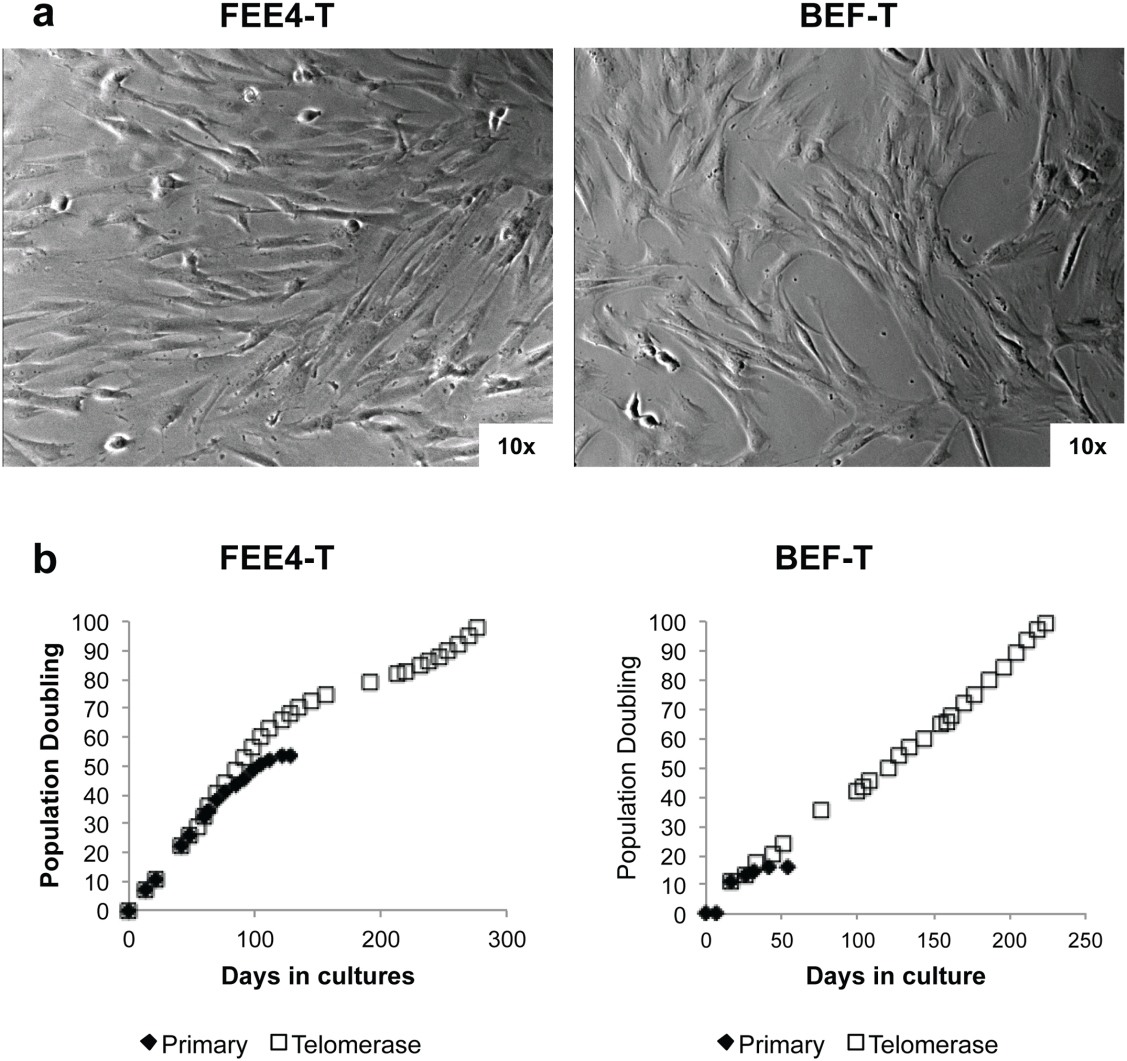
Telomerase-immortalized esophageal fibroblast cell line characterization. (a) FEE4-T and BEF-T exhibit spindle-like morphology. (b) Growth curves of telomerase-immortalized fibroblasts and primary cultures.

**Fig 2:**
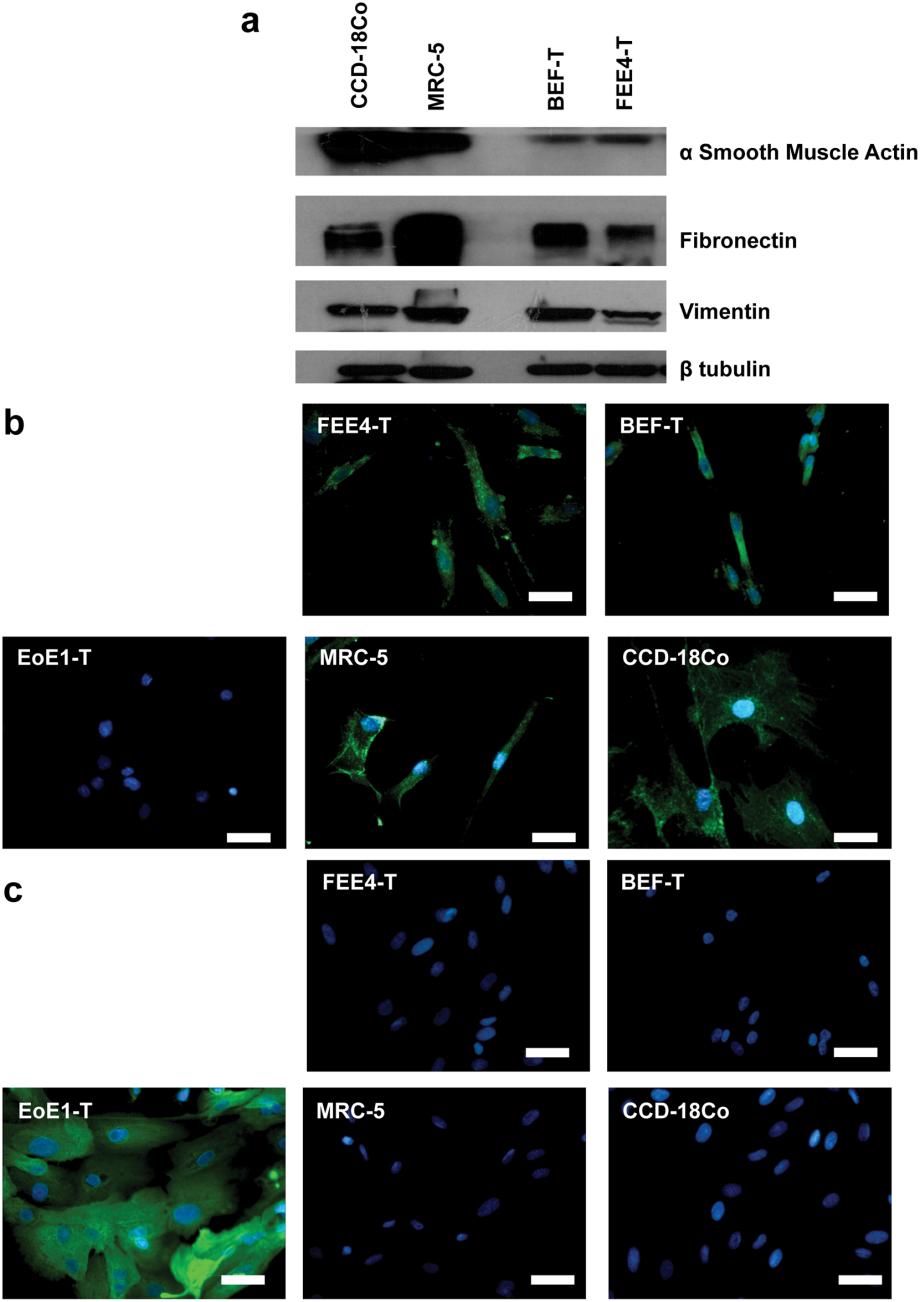
FEE4-T and BEF-T are esophageal fibroblasts. FEE4-T and BEF-T express fibroblast markers (a) α smooth muscle actin, fibronectin, and vimentin and (b) fibroblast surface protein (green). (c) Epithelial cells EoE1-T express epithelial marker pan-cytokeratin (green). Like colon fibroblasts (CCD-18Co) and lung fibroblasts (MRC-5), FEE4-T and BEF-T do not express pan-cytokeratin. CCD-18Co and MRC-5, fibroblast controls; EoE1-T, epithelial control. Nuclei were counterstained with DAPI. Scale bar = 50μm.

Fibroblasts were isolated from esophageal biopsy specimens. The growth of primary cultures of the esophageal fibroblasts reached senescence at ~50 population doublings for FEE4 and ~20 population doublings for BEF. Telomerase-immortalized esophageal fibroblast cell lines FEE4-T and BEF-T grown on plastic substrates exhibited spindle-like morphology (Fig 1a) and continued to grow beyond 100 population doublings (Fig 1b) unlike the primary cultures. The population doubling times of FEE4-T and BEF-T were approximately 60 and 56 hours, respectively. The TRAP-eze detection kit confirmed substantial telomerase activity after the introduction of human telomerase reverse transcriptase (data not shown). Both cell lines, like colon fibroblasts (CCD-18Co) and lung fibroblasts (MRC-5), expressed fibroblast markers such as α smooth muscle actin, fibronectin, vimentin by Western blot analysis (Fig 2a) and fibroblast surface protein by immunofluorescence staining (Fig 2b). Additionally, the two cell lines did not express pan-cytokeratin, an epithelial cell marker, confirming that they are indeed esophageal fibroblasts (Fig 2c). The fibroblast telomerase-immortalized cell lines did not exhibit signs of malignant transformation as evidenced by anchorage-dependent growth in soft agar, appropriate p53 response to UV-induced DNA damage, and karyotype by conventional cytogenetic analysis (data not shown). [27–29, 33, 34] These fibroblasts adequately contracted collagen matrices that were suitable for 3D organotypic cultures with esophageal epithelial cells EoE2-T (S1 Fig).

### Th2 Cytokines Induce Esophageal Fibroblasts to Express Eotaxin-3 via STAT6 Signaling

**Fig 3:**
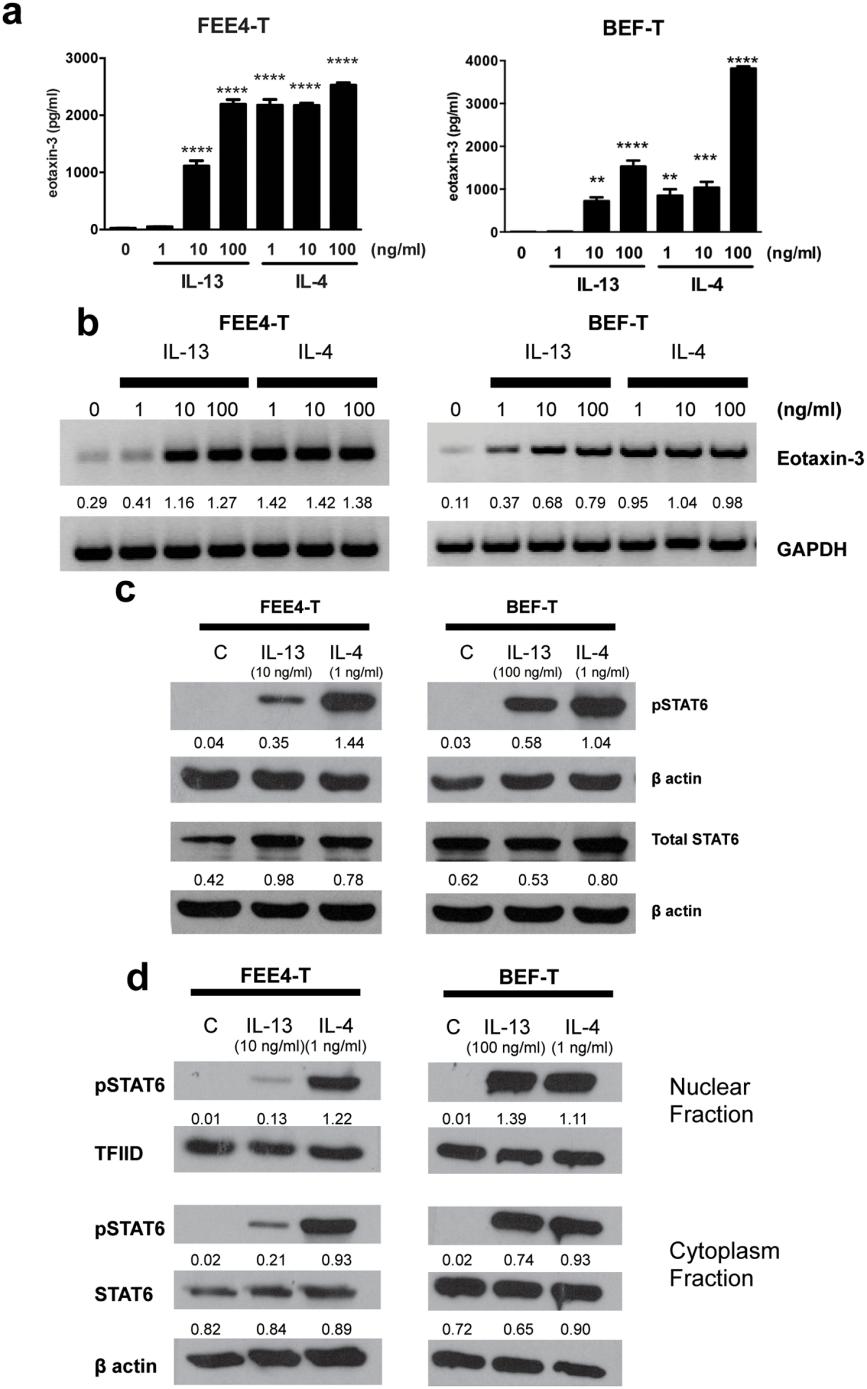
Th2 cytokines induce eotaxin-3 expression and activate STAT6 in esophageal fibroblasts. IL-13 and IL-4 induce (a) eotaxin-3 protein secretion, (b) eotaxin-3 mRNA expression, (c) STAT6 phosphorylation and (d) nuclear translocation of pSTAT6 in FEE4-T and BEF-T cells. Data are the means ± SEM. *p<0.05, **p<0.01, ***p<0.001, ****p<0.0001 compared to unstimulated cells. Densitometry values are normalized to their respective loading controls (GAPDH, β actin, or TFIID). C, control; pSTAT6, phosphorylated STAT6.

To investigate how fibroblasts might contribute to the pathogenesis of EoE, we studied Th2 cytokine effects on eotaxin-3 expression in FEE4-T and BEF-T cells. At baseline, FEE4-T and BEF-T secreted little eotaxin-3 protein. However, when treated with IL-13 or IL-4 at varying concentrations (1–100 ng/ml) for 48 hours, both fibroblast cell lines exhibited a substantial increase in eotaxin-3 secretion (Fig 3a). IL-13 at concentrations ≥10 ng/ml significantly increased eotaxin-3 protein secretion in FEE4-T and BEF-T. IL-4 at concentrations ≥1 ng/ml significantly increased eotaxin-3 protein secretion in both cell lines. Similarly, primary cultures of FEE4 fibroblasts demonstrated robust eotaxin-3 secretion in response to IL-13 and IL-4 stimulation (S2 Fig). There were no remaining primary BEF cells available to examine their response to Th2 cytokine stimulation. We then used RT-PCR to determine whether Th2 cytokines (IL-13 and IL-4) upregulate eotaxin-3 mRNA expression in esophageal fibroblasts. Both IL-13 (1–100 ng/ml) and IL-4 (1–100 ng/ml) strongly induced upregulation of eotaxin-3 mRNA within 48 hours of stimulation (Fig 3b).

The transcription of eotaxin-3 is regulated by the transcription factor STAT6 in epithelial cells.[2, 35] We performed Western blotting for phosphorylated STAT6 and total STAT6 to determine whether Th2 cytokines activate STAT6 in fibroblasts. IL-13 (10–100 ng/ml) and IL-4 (1 ng/ml) stimulation phosphorylated STAT6 in both fibroblast cell lines within 10 minutes (Fig 3c). Separation of the nuclear fraction from the cytoplasm fraction revealed that phosphorylated STAT6 translocated into the nucleus within 20 minutes of stimulation (Fig 3d).

### Omeprazole Does Not Block Eotaxin-3 Expression in Esophageal Fibroblasts

**Fig 4:**
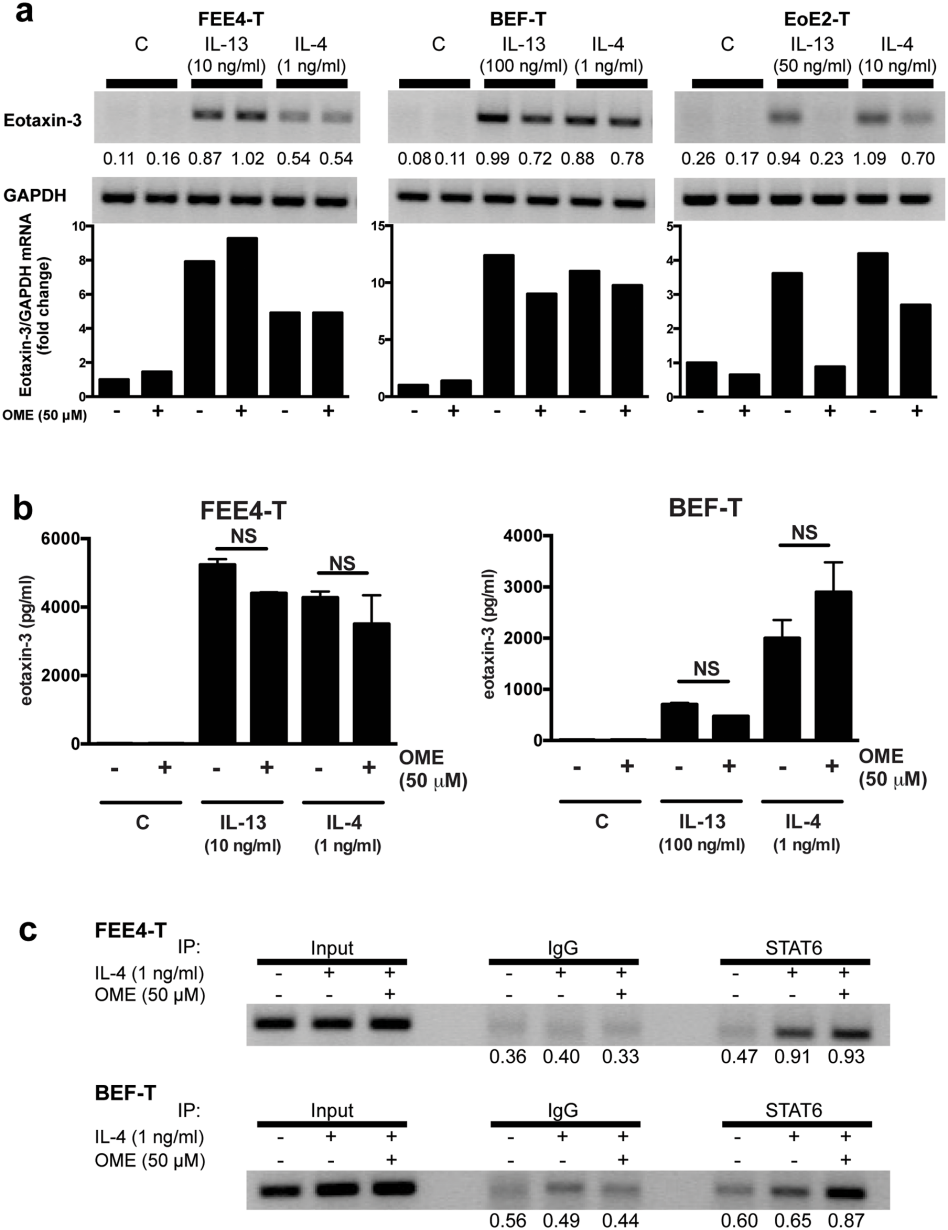
Omeprazole does not block Th2 cytokine-stimulated eotaxin-3 expression in esophageal fibroblasts. (a) Treatment with omeprazole (OME) does not substantially decrease Th2 cytokine-induced eotaxin-3 mRNA expression at 48 hours in esophageal fibroblast cell lines FEE4-T and BEF-T when compared to esophageal epithelial cell line EoE2T. Graph depicts the fold change in eotaxin-3/GAPDH mRNA levels for the representative gels. (b) Treatment with omprazole does not significantly decrease protein secretion at 48 hours in esophageal fibroblast cell lines. (c) Omeprazole does not interfere with IL-4-induced STAT6 binding to the eotaxin-3 promoter in esophageal fibroblast cell lines. Densitometry values are normalized to loading control (GAPDH or Input Fraction). Data are the means ± SEM. NS, not significant.

In earlier studies in esophageal squamous epithelial cells, we showed that PPIs block STAT6 from binding the eotaxin-3 promoter, thus inhibiting Th2-cytokine-induced eotaxin-3 mRNA and protein expression up to 48 hours.[2, 3] We examined whether eotaxin-3 expression by esophageal fibroblasts also can be blocked by PPIs. Esophageal fibroblasts were stimulated with IL-13 (10–100 ng/ml) or IL-4 (1 ng/ml) in the presence or absence of omeprazole. Unlike in esophageal epithelial cells, omeprazole did not substantially reduce eotaxin-3 mRNA levels after Th2 cytokine stimulation of our esophageal fibroblasts at 48 hours (Fig 4a) or at earlier time points (data not shown). In addition, omeprazole had no significant effect on eotaxin-3 protein secretion in Th2 cytokine-stimulated fibroblasts after 48 hours (Fig 4b), whereas omeprazole maintained suppressive effects in epithelial cells (S3 Fig). In esophageal epithelial cells, we have previously shown that omeprazole blocks the transcription factor STAT6 from binding the eotaxin-3 promoter.[3] However, in esophageal fibroblasts, omeprazole failed to block STAT6 binding to the eotaxin-3 promoter (Fig 4c).

### JAK-STAT6 Inhibitors Block Eotaxin-3 Expression in Esophageal Fibroblasts and Esophageal Epithelial Cells

**Fig 5:**
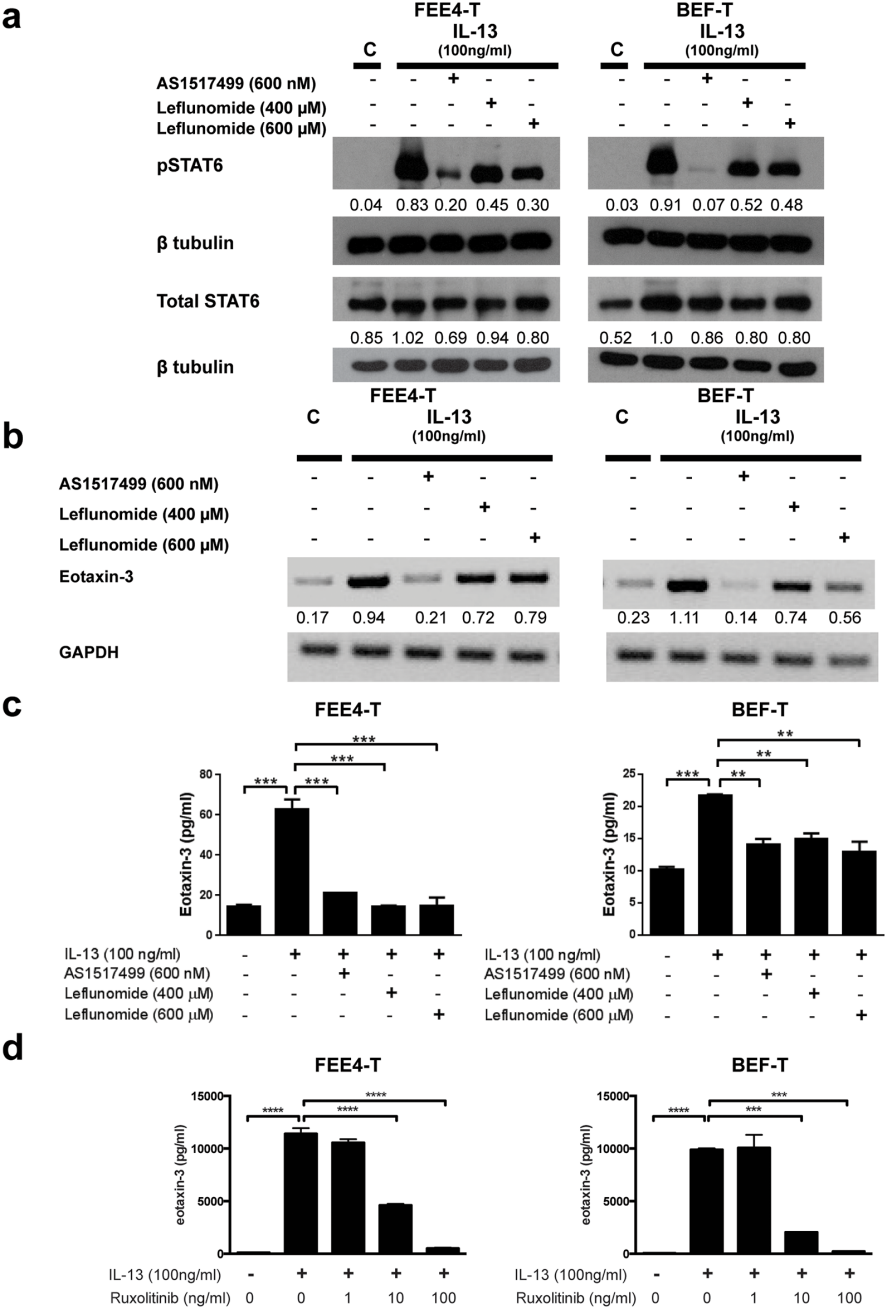
JAK-STAT6 inhibitors decrease IL-13-induced eotaxin-3 expression in esophageal fibroblasts. IL-13-induced (a) STAT6 phosphorylation, (b) eotaxin-3 mRNA, and (c) eotaxin-3 protein expression are decreased in esophageal fibroblasts, following treatment with JAK-STAT6 inhibitors (AS1517499 and leflunomide). (d) Selective JAK1/2 inhibitor ruxolitinib blocked IL-13-induced eotaxin-3 protein secretion. Densitometry values are normalized to their respective loading controls (β tubulin or GAPDH). Data are the means ± SEM. *p<0.05, **p<0.01, ***p<0.001, ****p<0.0001.

Since STAT6 regulates eotaxin-3 expression, we hypothesized that STAT6 might be a promising target for the treatment of EoE. To explore this hypothesis, we used AS1517499, a small molecule that has demonstrated selective inhibition of STAT6.[36] We also used the clinically approved drug leflunomide, which is used for treating rheumatoid arthritis. Leflunomide is a tyrosine kinase inhibitor that has demonstrated inhibitory effects on STAT6 tyrosine phosphorylation.[37, 38] In preliminary studies, esophageal fibroblasts remained healthy when exposed to AS1517499 at various doses and durations. When exposed to leflunomide for 24 hours, the fibroblasts exhibited cell stress and death (data not shown). Therefore, leflunomide exposure was limited to 6 hours, during which the fibroblasts were still healthy-appearing. In our esophageal fibroblasts, both AS1517499 (600 nM) and leflunomide (400 and 600 μM) markedly suppressed phosphorylation of STAT6 within 1 hour of IL-13 stimulation (Fig 5a). At 3 and 6 hours, AS1517499 and leflunomide also suppressed IL-13-stimulated phosphorylation of STAT6 in BEF-T (S4 Fig). AS1517499 markedly decreased eotaxin-3 mRNA transcription in both FEE4-T and BEF-T within 3 hours of IL-13 stimulation. Leflunomide decreased IL-13-induced eotaxin-3 mRNA transcription in FEE4-T and BEF-T, but to a lesser extent than AS1517499 (Fig 5b). At 1 hour, AS1517499 and leflunomide suppressed IL-13-stimulated eotaxin-3 mRNA expression in BEF-T. By 6 hours, IL-13-stimulated eotaxin-3 mRNA expression returned to baseline in cells treated with 400 μM of leflunomide, but remained suppressed in those treated with AS1517499 or with 600 μM of leflunomide (S5 Fig). Both AS1517499 and leflunomide significantly decreased eotaxin-3 protein secretion in the fibroblast cell lines within 6 hours of IL-13 stimulation (Fig 5c). The finding of tyrosine kinase inhibition on IL-13-induced eotaxin-3 protein secretion was further confirmed with a selective JAK1/2 inhibitor ruxolitinib at doses of 10 and 100 ng/ml for up to 48 hours (Fig 5d).

**Fig 6:**
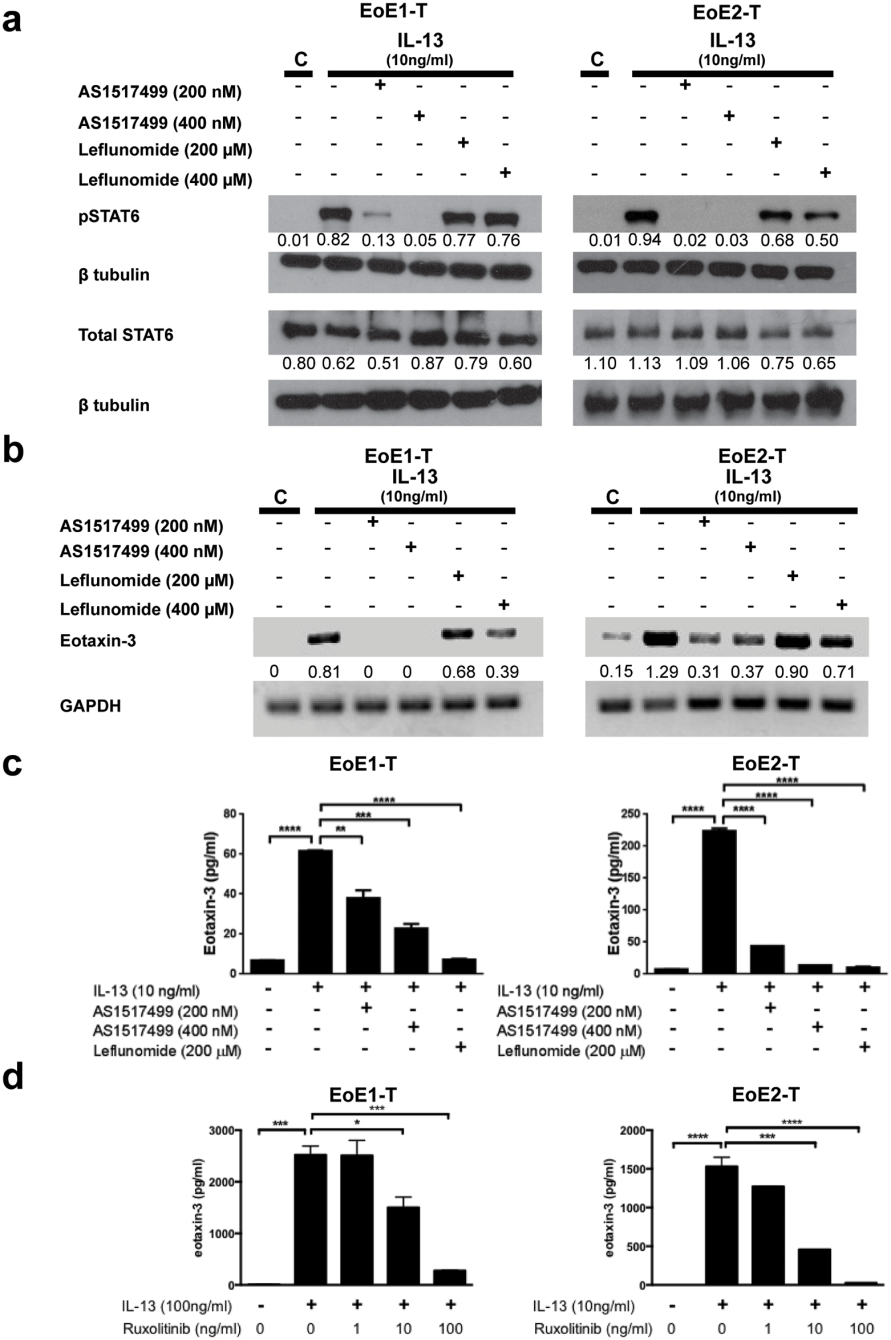
JAK-STAT6 inhibitors decrease IL-13-induced eotaxin-3 expression in esophageal epithelial cells. IL-13-induced (a) STAT6 phosphorylation, (b) eotaxin-3 mRNA, and (c) eotaxin-3 protein expression are decreased in esophageal epithelial cells, following treatment with JAK-STAT6 inhibitors (AS1517499 and leflunomide). (d) Selective JAK1/2 inhibitor ruxolitinib blocked IL-13-induced eotaxin-3 protein secretion. Densitometry values are normalized to their respective loading controls (β tubulin or GAPDH). Data are the means ± SEM. *p<0.05, **p<0.01, ***p<0.001, ****p<0.0001.

We also studied effects of the JAK-STAT6 inhibitors on esophageal epithelial cells using our previously established and well-characterized cell lines, EoE1-T and EoE-2. Similar to our fibroblast cell lines, EoE1-T and EoE2-T remained healthy when exposed to AS1517499, but exhibited cell stress with prolonged exposure to leflunomide. AS1517499 (200 and 400 nM) nearly abolished phosphorylation of STAT6 within 1 hour of IL-13 stimulation in both EoE1-T and EoE2-T. Leflunomide (200 and 400 μM) mildly decreased phosphorylation of STAT6 in the epithelial cells (Fig 6a). AS1517499 eliminated and markedly decreased eotaxin-3 transcription in EoE1-T and EoE2-T, respectively, within 3 hours of IL-13 stimulation. Leflunomide (400 μM) also decreased eotaxin-3 transcription (Fig 6b). Both inhibitors significantly decreased eotaxin-3 protein secretion within 6 hours of IL-13 stimulation in both EoE cell lines (Fig 6c). Lastly, the selective JAK1/2 inhibitor ruxolitinib at doses of 10 and 100 ng/ml significantly decreased IL-13-induced eotaxin-3 protein secretion for up to 48 hours (Fig 6d).

## Discussion

We have developed new human, telomerase-immortalized, esophageal fibroblast cell lines. They express typical fibroblast markers, exhibit no evidence of malignant transformation, and are suitable for organotypic cultures with esophageal epithelial cells. We have demonstrated that, as in esophageal epithelial cells, Th2 cytokines cause esophageal fibroblasts to express eotaxin-3 via STAT6 signaling. Unlike esophageal epithelial cells, however, the PPI omeprazole does not block Th2 cytokine-stimulated eotaxin-3 expression in esophageal fibroblasts. This suggests that PPIs might have little effect on esophageal subepithelial inflammation and fibrosis. Finally, we have demonstrated that JAK-STAT6 pathway inhibitors block Th2 cytokine-induced eotaxin-3 expression in esophageal fibroblasts as well as in esophageal epithelial cells. Thus, JAK-STAT6 inhibitors might have a therapeutic role in alleviating both the epithelial inflammation and subepithelial fibrosis in eosinophilic esophagitis.

Earlier studies have focused on epithelial cells and their role in epithelial inflammation in EoE. However, subepithelial fibrosis also is clinically important and has been difficult to study largely due to the inaccessibility of the subepithelial layers that are beyond the reach of endoscopic biopsy forceps. We have established esophageal fibroblast cell lines in order to study molecular mechanisms of esophageal fibrogenesis in EoE. Although eotaxin-3 itself may not be directly fibrogenic, eosinophils drawn into the lamina propria by fibroblasts that produce eotaxin-3 can release fibrogenic factors such as transforming growth factor (TGF)β1 and major basic protein.[39] EoE is a Th2 cytokine-driven disorder, and we have demonstrated that esophageal fibroblasts *in vitro* respond to Th2 cytokine stimulation with robust expression of the eosinophil chemoattractant eotaxin-3. We have found that Th2 cytokine-induced expression of eotaxin-3 by esophageal fibroblasts is mediated through STAT6 signaling, just as it is in esophageal epithelial cells.[2] While we have previously seen inhibition of eotaxin-3 expression in epithelial cells by omeprazole, which blocks STAT6 binding to the eotaxin-3 promoter, this mechanism of inhibition did not occur in esophageal fibroblasts. [3] Thus, while PPIs might be effective at decreasing eosinophilia in the epithelium, our findings suggest that PPIs might have limited impact on subepithelial inflammation and fibrosis.

Addressing inflammation and remodeling in layers deeper than the epithelium is crucial because EoE is a transmural disease, and the serious clinical consequences of EoE are due to subepithelial remodeling. [4, 9] Therefore, we sought to block STAT6 signaling and explored the effect of JAK-STAT6 pathway inhibitors. We found that they decreased cytokine-stimulated eotaxin-3 expression in fibroblasts as well as in epithelial cells. Overall, the STAT6 selective inhibitor AS1517499 exhibited less cellular toxicity and better inhibition of eotaxin-3 expression than the tyrosine kinase inhibitor leflunomide. However, AS1517499 is a relatively novel agent and has only demonstrated efficacy in preclinical studies thus far.[40] Although leflunomide is a clinically approved drug used for treating rheumatoid arthritis, our cells *in vitro* displayed considerable evidence of toxicity and stress when treated at high doses or long duration with this agent. On the other hand, cells treated with ruxolitinib, a Janus kinase inhibitor with selectively for JAK1 and JAK2, for up to 48 hours, demonstrated inhibition of eotaxin-3 secretion without signs of cell toxicity and stress. Currently, ruxolitinib is clinically approved for treating patients with myelofibrosis and has been investigated for treatment of cutaneous diseases such as plaque psoriasis and alopecia areata. [41] While our study provides proof-of-concept that JAK-STAT6 inhibitors might have a role in the treatment of EoE, further studies are needed to optimize treatment agents, dosages and durations.

In conclusion, using non-neoplastic, telomerase-immortalized esophageal fibroblast cell lines that we developed from human esophageal biopsy specimens, we have shown that esophageal fibroblasts respond to Th2 cytokine stimulation by expressing eotaxin-3 through STAT6 signaling. Although this response to Th2 cytokines is similar to that of esophageal epithelial cells, omeprazole does not block eotaxin-3 expression in esophageal fibroblasts as it does in epithelial cells. Thus, PPIs might be effective at decreasing eosinophilia in the epithelium, but our findings suggest that PPIs will likely have limited impact on subepithelial processes such as fibrosis. On the other hand, our finding that JAK-STAT6 inhibitors block Th2 cytokine-induced eotaxin-3 expression in esophageal fibroblasts as well as in esophageal epithelial cells suggests a potential therapeutic role for these agents in treating both the epithelial inflammation and subepithelial fibrosis in eosinophilic esophagitis.

## Supporting Information

S1 Fig Esophageal fibroblasts in 3D organotypic cultures. Esophageal fibroblasts BEF-T formed contracted collagen matrices allowing esophageal epithelial cells EoE2-T to layer on top forming an epithelial layer.
(DOCX)

S2 Fig Th2 cytokines induce eotaxin-3 protein secretion in primary esophageal fibroblasts. Primary FEE4 fibroblasts increased their secretion of eotaxin-3 protein with IL-13 (10–100 ng/ml) and IL-14 (1–100 ng/ml) stimulation. Data are the means ± SEM. **p<0.01, ***p<0.001, ****p<0.0001 compared to unstimulated cells (one-way ANOVA and Dunnett multiple comparison).
(DOCX)

S3 Fig Omeprazole blocks IL-13-stimulated eotaxin-3 protein secretion in epithelial cells, not in fibroblasts. Omeprazole blocks IL-13-stimulated eotaxin-3 protein secretion up to 48 hours in epithelial cells EoE2-T, while not in fibroblasts BEF-T. Data are the means ± SEM. *p<0.05, **p<0.01, ****p<0.0001 compared to untreated, and ++++p<0.0001 (one-way ANOVA and Bonferroni multiple comparison).
(DOCX)

S4 Fig JAK-STAT6 inhibitors suppress IL-13-stimulated phosphorylation of STAT6 in BEF-T. AS1517499 suppresses IL-13-stimulated phosphorylation of STAT6 at 3 and 6 hours. Leflunomide, at both concentrations, suppresses IL-13-stimulated phosphorylation of STAT6 at 3 and 6 hours.
(DOCX)

S5 Fig JAK-STAT6 inhibitors suppress IL-13-stimulated eotaxin-3 mRNA expression in BEF-T. AS1517499 suppresses IL-13-stimulated eotaxin-3 mRNA expression at 1 and 6 hours. Leflunomide, at both concentrations, suppresses IL-13-stimulated eotaxin-3 mRNA expression at 1 hour. By 6 hours, IL-13-stimulated eotaxin-3 mRNA levels returned to baseline in cells treated with the 400 μM dose of leflunomide, but remained suppressed in those treated with AS1517499 or with the 600 μM dose of leflunomide.
(DOCX)

## Abbreviations

DAPI: 4’, 6-diamidino-2-phenylindole
ELISA: enzyme-linked immunosorbent assay
EoE: eosinophilic esophagitis
GERD: gastro-esophageal reflux disease
GAPDH: glyceraldehyde-3-phosphate dehydrogenase
IL: interleukin
JAK: Janus kinase
OME: omeprazole
PCR: polymerase chain reaction
PPI: proton pump inhibitor
PPI-REE: proton pump inhibitor responsive esophageal eosinophilia
STAT: signal transducer and activator of transcription
SEM: standard error of the mean
Th: T helper
