# Supplementary material for: JAK-STAT6 Pathway Inhibitors Block Eotaxin-3 Secretion by Epithelial Cells and Fibroblasts from Esophageal Eosinophilia Patients: Promising Agents to Improve Inflammation and Prevent Fibrosis in EoE
Source: PLoS One. 2016 Jun 16;11(6):e0157376. doi: 10.1371/journal.pone.0157376 (PMC4911010; doi:10.1371/journal.pone.0157376)
Supplement: S1 Fig — Esophageal fibroblasts BEF-T formed contracted collagen matrices allowing esophageal epithelial cells EoE2-T to layer on top forming an epithelial layer. (DOCX) [file pone.0157376.s001.docx]

**S1 Fig**

**S1 Fig. Esophageal fibroblasts in 3D organotypic cultures.**

Esophageal fibroblasts BEF-T formed contracted collagen matrices allowing esophageal epithelial cells EoE2-T to layer on top forming an epithelial layer.
