# Supplementary material for: JAK-STAT6 Pathway Inhibitors Block Eotaxin-3 Secretion by Epithelial Cells and Fibroblasts from Esophageal Eosinophilia Patients: Promising Agents to Improve Inflammation and Prevent Fibrosis in EoE
Source: PLoS One. 2016 Jun 16;11(6):e0157376. doi: 10.1371/journal.pone.0157376 (PMC4911010; doi:10.1371/journal.pone.0157376)
Supplement: S2 Fig — Primary FEE4 fibroblasts increased their secretion of eotaxin-3 protein with IL-13 (10–100 ng/ml) and IL-14 (1–100 ng/ml) stimulation. Data are the means ± SEM. **p<0.01, ***p<0.001, ****p<0.0001 compared to unstimulated cells (one-way ANOVA and Dunnett multiple comparison). (DOCX) [file pone.0157376.s002.docx]

**S2 Fig**

**S2 Fig. Th2 cytokines induce eotaxin-3 protein secretion in primary esophageal fibroblasts.**

Primary FEE4 fibroblasts increased their secretion of eotaxin-3 protein with 48 hours of IL-13 (10-100 ng/ml) and IL-14 (1-100 ng/ml) stimulation. Data are the means ± SEM. **p<0.01, ***p<0.001, ****p<0.0001 compared to unstimulated cells (one-way ANOVA and Dunnett multiple comparison).
