# Supplementary material for: JAK-STAT6 Pathway Inhibitors Block Eotaxin-3 Secretion by Epithelial Cells and Fibroblasts from Esophageal Eosinophilia Patients: Promising Agents to Improve Inflammation and Prevent Fibrosis in EoE
Source: PLoS One. 2016 Jun 16;11(6):e0157376. doi: 10.1371/journal.pone.0157376 (PMC4911010; doi:10.1371/journal.pone.0157376)
Supplement: S3 Fig — Omeprazole blocks IL-13-stimulated eotaxin-3 protein secretion up to 48 hours in epithelial cells EoE2-T, while not in fibroblasts BEF-T. Data are the means ± SEM. *p<0.05, **p<0.01, ****p<0.0001 compared to untreated, and ++++p<0.0001 (one-way ANOVA and Bonferroni multiple comparison). (DOCX) [file pone.0157376.s003.docx]

**S3 Fig**

**S3 Fig.** **Omeprazole blocks IL-13-stimulated eotaxin-3 protein secretion in epithelial cells, not in fibroblasts.**

Omeprazole blocks IL-13-stimulated eotaxin-3 protein secretion up to 48 hours in epithelial cells EoE2-T, while not in fibroblasts BEF-T. Data are the means ± SEM. *p<0.05, **p<0.01, ****p<0.0001 compared to untreated, and ^++++^p<0.0001 (one-way ANOVA and Bonferroni multiple comparison).
