# Supplementary material for: JAK-STAT6 Pathway Inhibitors Block Eotaxin-3 Secretion by Epithelial Cells and Fibroblasts from Esophageal Eosinophilia Patients: Promising Agents to Improve Inflammation and Prevent Fibrosis in EoE
Source: PLoS One. 2016 Jun 16;11(6):e0157376. doi: 10.1371/journal.pone.0157376 (PMC4911010; doi:10.1371/journal.pone.0157376)
Supplement: S4 Fig — AS1517499 suppresses IL-13-stimulated phosphorylation of STAT6 at 3 and 6 hours. Leflunomide, at both concentrations, suppresses IL-13-stimulated phosphorylation of STAT6 at 3 and 6 hours. (DOCX) [file pone.0157376.s004.docx]

**S4 Fig**

**S4 Fig. JAK-STAT6 inhibitors suppress IL-13-stimulated phosphorylation of STAT6 in BEF-T.**

AS1517499 suppresses IL-13-stimulated phosphorylation of STAT6 at 3 and 6 hours. Leflunomide, at both concentrations, suppresses IL-13-stimulated phosphorylation of STAT6 at 3 and 6 hours.
