# Supplementary material for: JAK-STAT6 Pathway Inhibitors Block Eotaxin-3 Secretion by Epithelial Cells and Fibroblasts from Esophageal Eosinophilia Patients: Promising Agents to Improve Inflammation and Prevent Fibrosis in EoE
Source: PLoS One. 2016 Jun 16;11(6):e0157376. doi: 10.1371/journal.pone.0157376 (PMC4911010; doi:10.1371/journal.pone.0157376)
Supplement: S5 Fig — AS1517499 suppresses IL-13-stimulated eotaxin-3 mRNA expression at 1 and 6 hours. Leflunomide, at both concentrations, suppresses IL-13-stimulated eotaxin-3 mRNA expression at 1 hour. By 6 hours, IL-13-stimulated eotaxin-3 mRNA levels returned to baseline in cells treated with the 400 μM dose of leflunomide, but remained suppressed in those treated with AS1517499 or with the 600 μM dose of leflunomide. (DOCX) [file pone.0157376.s005.docx]

**S5 Fig**

**S5 Fig. JAK-STAT6 inhibitors suppress IL-13-stimulated eotaxin-3 mRNA expression in BEF-T.**

AS1517499 suppresses IL-13-stimulated eotaxin-3 mRNA expression at 1 and 6 hours. Leflunomide, at both concentrations, suppresses IL-13-stimulated eotaxin-3 mRNA expression at 1 hour. By 6 hours, IL-13-stimulated eotaxin-3 mRNA levels returned to baseline in cells treated with the 400 μM dose of leflunomide, but remained suppressed in those treated with AS1517499 or with the 600 μM dose of leflunomide.
